# Cataract surgery in patients with complex conditions

**Published:** 2019-02-10

**Authors:** Wanjiku Mathenge

**Affiliations:** 1 Consultant Ophthalmologist and Director of Training and Research: Rwanda National Institute of Ophthalmology and Dr Agarwal's Eye Hospital, Kigali, Rwanda.

**Cataract surgery is not always straightforward, but with careful planning by the surgical team, patients with complex conditions can still have a successful outcome.**

**Figure:**
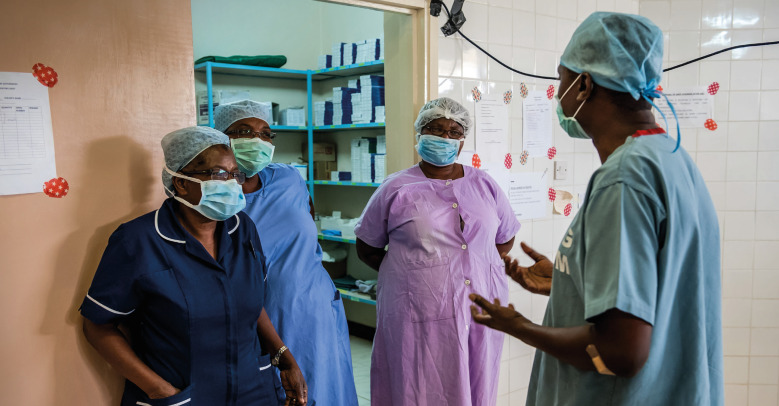
Nurses being briefed before a cataract operation. **MALAWI** Rachel Palmer/Sightsavers

Every ophthalmologist encounters patients with challenging or complicated cataract presentation from time to time, which tests our surgical skills and ability to manage difficult circumstances. The challenge may come from zonular weakness; cataract in very young patients; cataract with corneal opacities; cataract with co-morbidities, such as uveitis, glaucoma, diabetic eye disease or age-related macular degeneration; or intraoperative complications, such as posterior capsule tears and zonule or iris dialyses.

As surgeons, we must rely on our surgical skills, intelligent decision making, and the numerous new technologies that have revolutionised our ability to not just complete the operation, but achieve the best outcome possible.

When I encounter a challenging case, I rely on the following generic guiding principles, which I have learnt from my mentors and from experience, on how best to optimise surgical outcomes in these patients.

- Know your own surgical limitations and refer to a more experienced surgeon when necessary.
- Be vigilant: recognise and anticipate challenges before surgery and ensure you have the correct tools in the operating theatre to help manage any issues that arise.
- Manage the basics to reduce the challenges you are facing. Dilate the pupil as widely as possible, use the appropriate anaesthetic technique, stain the capsule for more predictable capsulorrhexis, choose the right viscoelastic for difficult steps and use reliable instruments and microscopes.
- Develop skills using a range of techniques and technologies because every eye is different. For example, the use of capsular support systems, scleral fixation techniques, small pupil management techniques and vitreous management and optic capture techniques in paediatric cataract.
- Have a plan, and have a back-up plan. This helps the surgeon to stay calm, which keeps the patient calm. For example, anticipate poor pupil dilation in uveitic eyes or weak zonules in pseudoexfoliation, and plan for the worst.
- Manage inflammation and complications such as macular oedema as well as you can before and during surgery. This is important in patients with uveitis and diabetes, as well as those with ocular surface disease.
- Use the appropriate technology, or a combination of techniques, for each challenging case to improve surgical effectiveness and efficiency. Ask yourself the following questions: will there really be any added benefit in using a toric or multifocal IOL in this case? Would a combined cataract-glaucoma procedure produce better outcomes for this patient? Should I give an anti-VEGF injection at the time of surgery? Other techniques that help improve outcomes include scraping off the corneal epithelium to increase visibility when appropriate, and the use of adrenergic agents in eyes with floppy irises.
- Follow the correct postoperative regimen for challenging cases in order to improve outcomes. This may include good refractive management, long-term steroids after surgery for uveitic patients, or the use of non-steroidal anti-inflammatory drugs to prevent worsening macular oedema in patients with diabetes.

In conclusion, prepare yourself, your team and your patient for difficult cataract surgery. Always communicate known and expected challenges to the patient before surgery so that you set realistic expectations about the outcome. Discussing the appropriate postoperative care, especially where it is different from routine care, will then be easier.

